# Delayed Cryptochrome Degradation Asymmetrically Alters the Daily Rhythm in Suprachiasmatic Clock Neuron Excitability

**DOI:** 10.1523/JNEUROSCI.0691-17.2017

**Published:** 2017-08-16

**Authors:** Sven Wegner, Mino D.C. Belle, Alun T.L. Hughes, Casey O. Diekman, Hugh D. Piggins

**Affiliations:** ^1^Faculty of Biology, Medicine, and Health University of Manchester, Manchester, United Kingdom M13 9PT, and; ^2^Department Mathematical Sciences, New Jersey Institute of Technology, Newark, New Jersey 07102

**Keywords:** brain slice, circadian, cryptochrome, electrophysiology, fbxl3, post-translational modification, suprachiasmatic

## Abstract

Suprachiasmatic nuclei (SCN) neurons contain an intracellular molecular circadian clock and the Cryptochromes (CRY1/2), key transcriptional repressors of this molecular apparatus, are subject to post-translational modification through ubiquitination and targeting for proteosomal degradation by the ubiquitin E3 ligase complex. Loss-of-function point mutations in a component of this ligase complex, Fbxl3, delay CRY1/2 degradation, reduce circadian rhythm strength, and lengthen the circadian period by ∼2.5 h. The molecular clock drives circadian changes in the membrane properties of SCN neurons, but it is unclear how alterations in CRY1/2 stability affect SCN neurophysiology. Here we use male and female *Afterhours* mice which carry the circadian period lengthening loss-of-function *Fbxl3^Afh^* mutation and perform patch-clamp recordings from SCN brain slices across the projected day/night cycle. We find that the daily rhythm in membrane excitability in the ventral SCN (vSCN) was enhanced in amplitude and delayed in timing in *Fbxl3^Afh/Afh^* mice. At night, vSCN cells from *Fbxl3^Afh/Afh^* mice were more hyperpolarized, receiving more GABAergic input than their *Fbxl3*^+/+^ counterparts. Unexpectedly, the progression to daytime hyperexcited states was slowed by *Afh* mutation, whereas the decline to hypoexcited states was accelerated. In long-term bioluminescence recordings, GABA_A_ receptor blockade desynchronized the *Fbxl3*^+/+^ but not the *Fbxl3^Afh/Afh^* vSCN neuronal network. Further, a neurochemical mimic of the light input pathway evoked larger shifts in molecular clock rhythms in *Fbxl3^Afh/Afh^* compared with *Fbxl3*^+/+^ SCN slices. These results reveal unanticipated consequences of delaying CRY degradation, indicating that the *Afh* mutation prolongs nighttime hyperpolarized states of vSCN cells through increased GABAergic synaptic transmission.

**SIGNIFICANCE STATEMENT** The intracellular molecular clock drives changes in SCN neuronal excitability, but it is unclear how mutations affecting post-translational modification of molecular clock proteins influence the temporal expression of SCN neuronal state or intercellular communication within the SCN network. Here we show for the first time, that a mutation that prolongs the stability of key components of the intracellular clock, the cryptochrome proteins, unexpectedly increases in the expression of hypoexcited neuronal state in the ventral SCN at night and enhances hyperpolarization of ventral SCN neurons at this time. This is accompanied by increased GABAergic signaling and by enhanced responsiveness to a neurochemical mimic of the light input pathway to the SCN. Therefore, post-translational modification shapes SCN neuronal state and network properties.

## Introduction

Post-translational modification plays key roles in shaping neuronal activity and function (Henley et al., 2014; Zhang et al., 2015; Baroncelli et al., 2016). A key exemplar of this is the brain's master circadian pacemaker of the hypothalamic suprachiasmatic nuclei (SCN). Here, several thousands of neurons contain an intracellular circadian clock in which transcriptional/translational feedback loops (TTFLs) provide a molecular representation of the external 24 h cycle (Hastings et al., 2014; Bedont and Blackshaw, 2015; Evans, 2016). Within the TTFL, the transcription factors BMAL1 and CLOCK activate the expression of the Period (*Per1/2*) and Cryptochrome (*Cry1/2*) genes. Subsequently, the PER1/2 and CRY1/2 proteins interact and translocate to the nucleus where they function to repress the actions of BMAL1 and CLOCK. This results in PER-CRY inhibiting their own transcription (Reppert and Weaver, 2002; Okamura, 2004; Partch et al., 2014). Clock proteins are targeted for proteosomal degradation by phosphorylation as well as ubiquitination and these are vital to the normal timekeeping function of circadian clocks. In mammals, F-box (Fbx) proteins are central to this and contribute to ubiquitination/targeting for proteosomal degradation via the Spk-Cullin/F-box protein (SCF) E3 ligase complex (Teixeira and Reed, 2013; Skaar et al., 2014). For example, recent research establishes that Fbxl3 plays a key role in the mammalian circadian clock, with SCF^Fbxl3^ directing the degradation of CRY1 and CRY2 (Busino et al., 2007). Interestingly, two different point mutations, *Fbxl3^Ovtm^* (Godinho et al., 2007; Siepka et al., 2007) and *Fbxl3^Afh^* (Godinho et al., 2007) result in loss-of-function in Fbxl3, thereby delaying CRY1/2 ubiquitination and degradation. In the SCN clock, as well as circadian clocks present in other brain sites and peripheral tissues, these actions of *Fbxl3^Ovtm^* and *Fbxl3^Afh^* slow circadian oscillations and prolong circadian period by up to 2.5 h (Godinho et al., 2007; Siepka et al., 2007; Guilding et al., 2013). In addition, the amplitude of the TTFL is reduced by these mutations (Godinho et al., 2007; Siepka et al., 2007; Anand et al., 2013) and in the case of *Fbxl3^Afh^*, the robustness of the SCN circadian clock is weakened (Guilding et al., 2013). These investigations indicate that Fbxl3-mediated ubiquitination and targeting of CRYs for proteosomal degradation have key period lengthening and other notable effects on the SCN clock (Hirano et al., 2016).

The TTFL targets the membrane properties of SCN neurons and drives a daily rhythm in neuronal excitability that is essential for coordination among SCN autonomous oscillators as well as the effective communication of circadian phase information to the rest of the brain and body (Brown and Piggins, 2007; Colwell, 2011). During the day, SCN neurons become progressively depolarized and hyperexcited, increasing their discharge of action potentials (APs) or becoming silent through depolarization blockade (Kuhlman and McMahon, 2006; Belle, 2015). At night, SCN neurons transition to more hyperpolarized states, reducing AP firing rate, with some cells becoming quiescent. Surprisingly, although there is considerable knowledge of the molecular actions of post-translational modification mechanisms in the SCN, very little is known about how they influence SCN neuronal activity. Here we address this important knowledge gap and use SCN brain slices from *Fbxl3^Afh/Afh^* mice to show that in neurons of both dorsal SCN (dSCN) and ventral SCN (vSCN) subregions, *Fbxl3^Afh^* delays the daily rhythm in SCN excitability. Additionally, at night, *Fbxl3^Afh/Afh^* vSCN neurons become unusually hyperpolarized. Indeed, *Fbxl3^Afh^* asymmetrically alters the dynamics of vSCN neuronal membrane excitability, slowing the progression to hyperexcited levels during the day and accelerating the subsequent decline to hyperpolarized levels at night. This emerges in *Fbxl3^Afh/Afh^* mice through reduced intrinsic regulation of SCN neuronal state and elevated signaling by GABA, a neurotransmitter found in most SCN neurons (Moore and Speh, 1993; Buijs et al., 1994; Albers et al., 2017). Subsequently GABA's contribution to SCN neuronal synchrony is altered and the *Fbxl3^Afh^* SCN exhibits enhanced resetting to a physiologically relevant excitatory input. These findings reveal how stabilization of CRY1/2 degradation has unanticipated consequences on the dynamics of SCN neurophysiology, from the single cell to the neuronal network.

## Materials and Methods

### 

#### 

##### Animal housing.

All experiments were performed in accordance with the UK Animals (Scientific Procedures) Act of 1986 using procedures approved by The University of Manchester Review Ethics Panel. Animals were group housed under a 12 h light/dark (LD) cycle. In LD conditions, lights-on was defined as Zeitgeber Time 0 (ZT0) and lights-off as ZT12. Food (Bekay, B&K Universal) and water were available *ad libitum*, temperature was maintained at 20 ± 2°C and humidity at ∼40%. Light intensity in the breeding colony was ∼45 μWcm^2^. Experiments were conducted on age- and sex-matched adult mice, using congenic *Fbxl3*^+/+^ and *Fbxl3^Afh/Afh^* littermates. Mice were derived from three breeding pairs of male *Fblxl3^Afh/Afh^* × female *Fbxl3*^*Afh*/+^ animals provided by M. Hastings, MRC Laboratory of Molecular Biology, Cambridge, UK. This strain was originally generated by P. Nolan at the Mammalian Genetics Unit, MRC Harwell, Oxfordshire, UK (Yoo et al., 2004). All original breeding stock had been backcrossed for 9–10 generations on the *mPer2::Luc* homozygous mouse background (Yoo et al., 2004). Mice were genotyped using an allelic discrimination assay as previously described (Godinho et al., 2007; Guilding et al., 2013). Consistent with our published work on this strain (Guilding et al., 2013), preliminary investigation indicated no obvious intragenotype sex differences in the experimental measures reported and subsequently data were combined for the purposes of analysis.

For experiments in which free-running behavior was assessed, animals were initially single-housed in running wheel-equipped cages under LD and then released into constant dark (DD). Under DD conditions, the onset of the wheel-running rhythm was defined as Circadian Time 12 (CT12). Running wheel activity data were acquired using Chronobiology Kit (Stanford Software Systems) and actograms created in Kit Analyze (Chronobiology Kit). Circadian period and amplitude were determined using χ^2^ periodogram in Kit Analyze (Chronobiology Kit).

##### Preparation of brain slices.

Mice were anesthetized with isoflurane (Abbott Laboratories) before cervical dislocation. Where indicated, animals were culled in complete darkness with the aid of night vision infrared goggles (Cobra Optics). After decapitation, the eyes were disconnected from the brain by cutting the optic nerve at the level of the eye ball. The lights were then switched on and the brain excised from the skull. Brains to be used in bioluminescence recordings were cooled and moistened with ice-cold HBSS (Sigma-Aldrich) supplemented with 0.035% sodium bicarbonate (Sigma-Aldrich), 0.01 m HEPES (Sigma-Aldrich) and 1 mg/ml penicillin-streptomycin (Invitrogen). Brains to be used for electrophysiology recordings were placed in ice-cold incubation artificial CSF (aCSF) containing the following (in mm): 95 NaCl, 1.8 KCl, 1.2 KH_2_PO_4_, 7 MgSO_4_, 26 NaHCO_3_, 15 glucose, 50 sucrose, 0.5 CaCl_2_, and phenol red 0.005 mg/L, pre-gassed with carbogen. Coronal slices (250 μm thick for both electrophysiological and bioluminescence recordings) were cut with a Campden 7000smz vibrating microtome (Campden Instruments).

##### Electrophysiological recordings.

**Figure 1.**
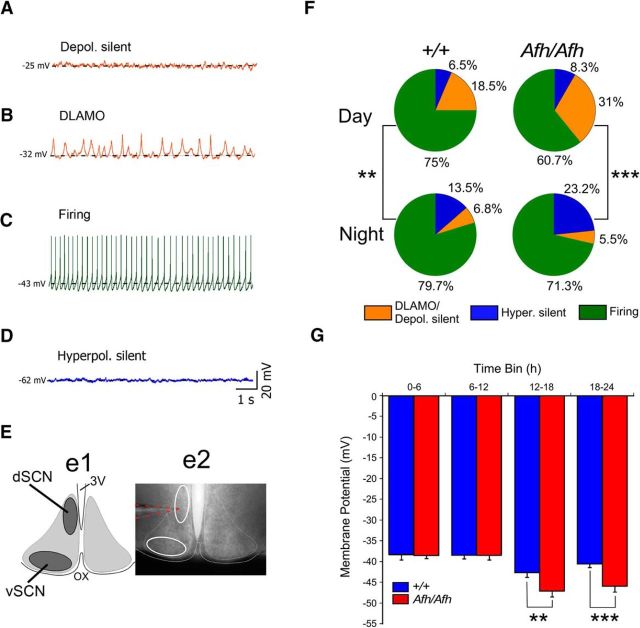
Reduced excitability of *Fbxl3^Afh/Afh^* vSCN neurons. ***A***–***D***, Four different electrophysiological states of SCN neurons were initially distinguished in both *Fbxl3*^+/+^ (+/+) and *Fbxl3^Afh/Afh^* (*Afh/Afh*) brain slices: (***A***) depolarized silent, (***B***) depolarized with low-amplitude membrane oscillations or DLAMO, (***C***) spontaneously firing, and (***D***) hyperpolarized silent. Broken horizontal black line in ***A***–***D*** delineates the resting membrane potential for the recording. ***E***, Recordings were made with pipettes visually guided into either the dSCN or vSCN depicted in the illustration (***Ee1***) that corresponds to delineated white-outlined ovals in the photomicrograph (***Ee2***) of live coronal SCN brain slice. 3V, Third ventricle; OX, optic chiasm. Broken orange lines highlight a recording pipette targeted at dSCN. ***F***, Percentages of cells in the different electrophysiological states (color coded as above, except cells in depolarized states ***A*** and ***B*** are collated (orange) for analysis as 1 category) during the day (top row) and night (bottom row) are shown as pie charts for +/+ and *Afh/Afh* genotypes. For the whole SCN, cell state was significantly associated with time of day for each genotype, with hyperpolarized states tending to be more numerous at night, particularly in the *Afh/Afh* SCN. ***G***, The mean RMP of *Fbxl3^Afh/Afh^* vSCN neurons was more hyperpolarized than that of +/+ vSCN neurons during the early (ZT12–ZT18) and later (ZT18–ZT24) night. No genotype differences were detected for RMP in the dSCN (data not shown). Data in ***G*** are plotted as mean ± SEM. **p < 0.01, ***p < 0.001.

Slices, containing the mid-coronal SCN section, were incubated for at least 1 h in pre-gassed recording aCSF containing the following (in mm): 127 NaCl, 1.8 KCl, 1.2 KH_2_PO_4_, 1.3 MgSO_4_, 26 NaHCO_3_, 15 glucose, 2.4 CaCl_2_, and phenol red 0.005 mg/L at room temperature. The aCSF was applied by gravity at a continuous flow of ∼3 ml/min. Visualization was performed by an Olympus BX51WI upright microscope with inbuilt infrared differential contrast optics mounted on a vibration-free air table (TMC). A Hitachi C106005 CCD camera system was used for visualization of the cells on a high-resolution black/white monitor. Photographs of the patch pipette were taken *in situ* for accurate anatomical documentation of the recorded neurons within the SCN. Aided in part by the shape of the optic chiasm and third ventricle as landmarks, recordings were made from the dorsal and ventral subregions of the SCN (dSCN and vSCN, respectively; Fig. 1*E*).

##### Current-clamp recordings.

Current-clamp data were acquired and analyzed as described previously (Belle et al., 2009). Briefly, recordings were performed with an npi BA-03X bridge amplifier (npi electronic) connected to a CED 1401 mk II A/D interface controlled by Spike2 software (Cambridge Electronic Design). Patch pipettes were filled with an intracellular solution containing the following (in mm): 130K-glutamate, 10 KCl, 2 MgCl_2_, 10 HEPES, 0.5 EGTA, 2 K_2_ATP, 0.5 NaGTP. To measure the membrane properties [input resistance (*R*_input_), firing pattern of the cell during/following excitatory or inhibitory stimuli] of the neurons in current-clamp mode, depolarizing (10–30 pA for 1 s) and hyperpolarizing (−10 to −30 pA for 500 ms) currents were used. Access resistance was typically ∼15 MΩ and series resistance ∼20 MΩ.

##### Synaptic current recordings.

Recordings of spontaneous postsynaptic currents (sPSCs) were performed as previously described (Belle et al., 2014). In brief, pipettes (4–6 MΩ) were filled with a solution identical to the one used for current-clamp recordings, except for 120 mm K-gluconate and 20 mm KCl. This [Cl^−^]_i_ causes the inhibitory sPSCs to reverse between −40 to −50 mV. At a holding potential of −70 mV, both GABA and glutamatergic PSCs appear as inward currents. To discriminate between glutamatergic and GABAergic PSCs, glutamatergic [d-2-amino-5-phosphonopentanoate (AP-5) and 6-cyano-7-nitroquinoxaline-2,3-dione (CNQX)] and GABAergic (gabazine) blockers were gravity applied in the bath solution. Gabazine, AP-5, and CNQX (Tocris Bioscience) were thawed from aliquots of concentrated stock solutions and diluted in aCSF to working concentrations on the day of the recording.

##### Electrophysiological data analysis.

Current-clamp data were analyzed with Spike2 software (versions 6 and 7, Cambridge Electronic Design). Passive membrane properties, such as resting membrane potential (RMP) and spontaneous firing rate (SFR) were determined within 2–5 min of establishing the whole-cell configuration, and before any current injections. Cells were removed from analysis if the RMP was unstable. The RMP was defined as the mean voltage over a 20 s window calculated using a custom-written Spike2 script (Sakhi et al., 2014). All cells included in the analysis were grouped in accordance to their spontaneous electrophysiological state: depolarized silent and depolarized low-amplitude membrane oscillations (DLAMO), firing and hyperpolarized silent. Depolarized silent and DLAMO cells were excluded from the SFR analysis because their level of membrane potential does not permit AP firing (Belle et al., 2009; Diekman et al., 2013). Because input signals can induce hyperpolarized silent cells to fire APs, they were included in the analysis of the SFR with a value of 0 Hz.

Voltage-clamp data analysis was done with pClamp v10 and Origin Pro v8.5 (OriginLab), software. Analysis of sPSC frequency was conducted off-line using template-based waveform sorting in Clampfit 10.2 (Molecular Devices). Only synaptic events with amplitudes >5 pA were used for analysis.

##### Bioluminescence recordings.

For bioluminescence recordings, 35 mm FluoroDishes (World Precision Instruments) were prepared with a layer of autoclaved high-vacuum grease (Dow Corning) on the top of the rim and loaded with 1.2 ml recording medium [3.5 g/L DMEM, d-glucose (Sigma-Aldrich), 0.035% sodium bicarbonate, 10 mm HEPES, 1 mg/ml penicillin-streptomycin, 5% B27, and 0.1 mm luciferin in autoclaved Milli-Q water] and a Millicell insert (Millipore) under sterile conditions. Tissue explants were microdissected from slices and placed on the Millicell insert. The dish was sealed by a glass coverslip. Prepared dishes were placed in a 37°C chamber and imaged with a self-contained Olympus LV200 luminescence microscopy system fitted with a cooled Hamamatsu C9100-13 EM-CCD camera using a 20× 0.45 NA LUCPLFLN objective (Olympus). Bright-field (bf) pictures were taken before and after recording of the bioluminescence signal in darkness. This was to reposition the slice following drug treatment and to confirm the regions-of-interest (ROIs). The recording parameters (EM: 4, gain: 1, exposure time: 1800 s) were identical for *Fbxl3*^+/+^ and *Fbxl3^Afh/Afh^* cultures. Treatments were performed by transferring the insert containing the tissue explant to a new culture dish containing fresh media with either vehicle or drug.

##### Bioluminescence data analysis.

Recorded images were transferred to tiff format and imported to ImageJ v1.37a (NIH). To analyze putative single cells over time, an ImageJ plugin (written in-house by Gareth Smith, University of Manchester, Manchester, UK) was used to select ROIs and trace minimal cell movements due to slice drift over the recording time of 8–10 d and after drug treatment. Eighteen to 22 cell-like ROIs were analyzed per slice and subregion (dSCN and vSCN). All bioluminescence data were then detrended by subtracting a 24 h running-average from the raw data and smoothed with a 3 h running average (Guilding et al., 2013). Peaks and nadirs were identified from those curves using the peak analysis function in Origin Pro v8.5 (OriginLab). Heatmaps were created using Microsoft Excel and the maximal color amplitude was normalized to the highest bioluminescence values for each individual ROI. The synchrony plots (*R* values) were calculated separately for each region and slice (18–22 cells/slice) using Rayleigh circular statistics and the mean ± SEM were calculated from those *R* values.

##### Experimental design and statistical analysis.

Behavior: assessment of wheel-running activity was performed using an independent groups design with genotype differences in circadian period and amplitude statistically determined using independent Student's *t* tests in KaleidaGraph v4.5 (Synergy Software) and significance set at *p* < 0.05. These are illustrated in Figure 3, where significance is denoted: *p < 0.05, ***p < 0.001. Current-clamp electrophysiology: to evaluate how the parameters (RMP, SFR, and *R*_input_) of different categories of cell varied by time of day, data were assigned to one of four 6 h time bins, depending on the time of day of recording. Subsequently, genotype and time of day effects and their potential interaction were evaluated by two-way ANOVA with planned single degree of freedom contrasts in JMP v11 (SAS Institute). To evaluate whether genotype, SCN subregion, and/or time of day influenced expression of SCN cell state, the proportions of cells in the different states were compared with the χ^2^ test using the online resource: . Pie charts and bar graphs were constructed using KaleidaGraph v4.5 (Synergy Software). Statistical significance in ANOVAs (including contrast tests) and χ^2^ test was defined as *p* < 0.05. A summary of these data are shown in Figure 1, where significance is denoted: **p < 0.01 and ***p < 0.001.

To compare the daily profiles of electrical properties in *Fbxl3*^+/+^ and *Fbxl3^Afh/Afh^* mice, penalized regression splines (Wood, 2006) were fit to smooth measurements of RMP, SFR, and *R*_input_ from SCN neurons sampled at ZTs throughout the projected LD cycle. The R package (R Foundation) “mgcv” was used to implement penalized cubic regression splines with four knots located at ZT = 0, 8, 16, and 24. Further, a cyclic spline basis was specified to ensure that the end points of the spline matched up because ZT 0 and 24 correspond to the same time of day. Genotype differences in the fitted splines were compared for RMP, *R*_input_, and SFR with the GLIMMIX procedure (SAS v9) with correction for multiple comparisons, and significance set at *p* < 0.05. These data are illustrated in Figure 3, with genotype differences indicated where the mean ± 95% confidence interval of each genotype's fitted cubic spline do not overlap.

For the frequency of GABAergic events recorded in voltage-clamp, data were statistically compared using independent Student's *t* tests with Bonferroni adjustment (and with Welch's correction in cases of unequal variance; Prism v7, GraphPad). Unless otherwise stated, *t* tests were two-tailed. All values are given as mean ± SEM. A summary of these data are shown in Figure 4, where the following are used to denote varying levels of statistical significance: **p < 0.001 for within genotype comparisons, #*p* < 0.05, and ##*p* < 0.01 for between-genotype comparisons.

For analysis of single-cell rhythms recorded with an EM-CCD camera, cell synchrony (*R* values) over the first three cycles in culture were statistically compared using two-way repeated-measures ANOVA and Sidak *post hoc* tests with significance set at *p* < 0.05 (Prism v7). To compare the effects of gabazine and vehicle treatment on *R* values on day 4 in culture, the Student's paired *t* test (Prism v7) was used with *p* < 0.05 considered as significant. For analysis of the actions of α-amino-3-hydroxy-5-methyl-4-isoxazolepropionic acid (AMPA) on luminometrically recorded rhythms, genotype and treatment effects were determined using Student's *t* tests (Prism v7). Summaries of the bioluminescence rhythms are shown in Figures 5 and 6, where the following are used to denote significance: *p < 0.05, **p < 0.01.

## Results

### Regional and phase-related reductions in the activity of Fbxl3^Afh/Afh^ SCN neurons

**Figure 2.**
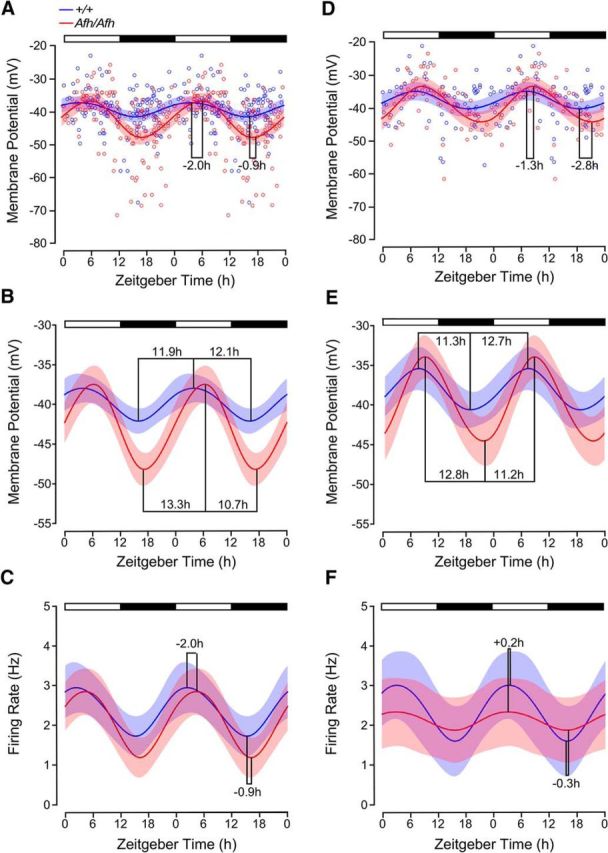
*Fbxl3^Afh^* mutation asymmetrically alters the daily rise and decline in membrane excitability of SCN neurons. The peak and nadir of resting membrane potential (***A***, ***B***) and spontaneous firing rate (***C***) of vSCN neurons were delayed by the *Afh* mutation. In the vSCN of *Fbxl3^Afh/Afh^* (*Afh/Afh*) mice, the daytime progression from nadir to peak resting membrane potential slowed (∼1.4 h; ***A***, ***B***), whereas the decline to the nocturnal nadir was accelerated (∼1.4 h). At night, the RMP was more hyperpolarized than that of *Fbxl3*^+/+^ vSCN neurons (+/+), whereas spontaneous firing rate did not differ between the genotypes (***C***). Prominent asymmetry in the daily increase and decrease in RMP was seen in the *Fbxl^Afh/Afh^* vSCN (***B***). Intergenotype comparison of the fitted spline with the GLIMMIX procedure indicated that RMP of *Afh/Afh* vSCN neurons differed from the RMP of +/+ SCN neurons across each ZT hour from ZT14–ZT22 (range *p* < 0.05–*p* < 0.001; ***A***, ***B***). Time of peak and nadir in firing rate were delayed by ∼2.0 and 0.9 h, respectively, in the *Afh/Afh* vSCN (***C***). Compared with the +/+ dSCN, time of maximal and minimal RMP were delayed by ∼1.3 and ∼2.8 h, respectively, in the *Afh/Afh* dSCN (***D***, ***E***). However, no intergenotype differences were detected in RMP of dSCN neurons across the projected day/night cycle (***D***, ***E***). The asymmetry in the daily increase and decrease in RMP seen in the *Fbxl3*^+/+^ dSCN was reversed in the dSCN of *Fbxl3^Afh/Afh^* mice (***E***). In the dSCN, the amplitude of the firing rate rhythm was reduced in the *Fbxl3^Afh/Afh^* compared with *Fbxl3*^+/+^ animals (0.4 vs 1.4 Hz), whereas the time of peak and nadir in firing rate of dSCN neurons showed very small genotype-related differences (***F***). Data in ***A*** and ***D*** are scatter plots of individual data points (*Afh/Afh* in red unfilled circles; +/+ in blue unfilled circles) fitted with cubic spline fits of the mean ± 95% confidence interval, whereas data in ***B***, ***C***, ***E***, and ***F*** are plotted as cubic spline fits of the mean ± 95% confidence interval. ***B*** and ***E*** are the cubic splines in ***A*** and ***D***, respectively, plotted against an expanded ordinate.

**Figure 3.**
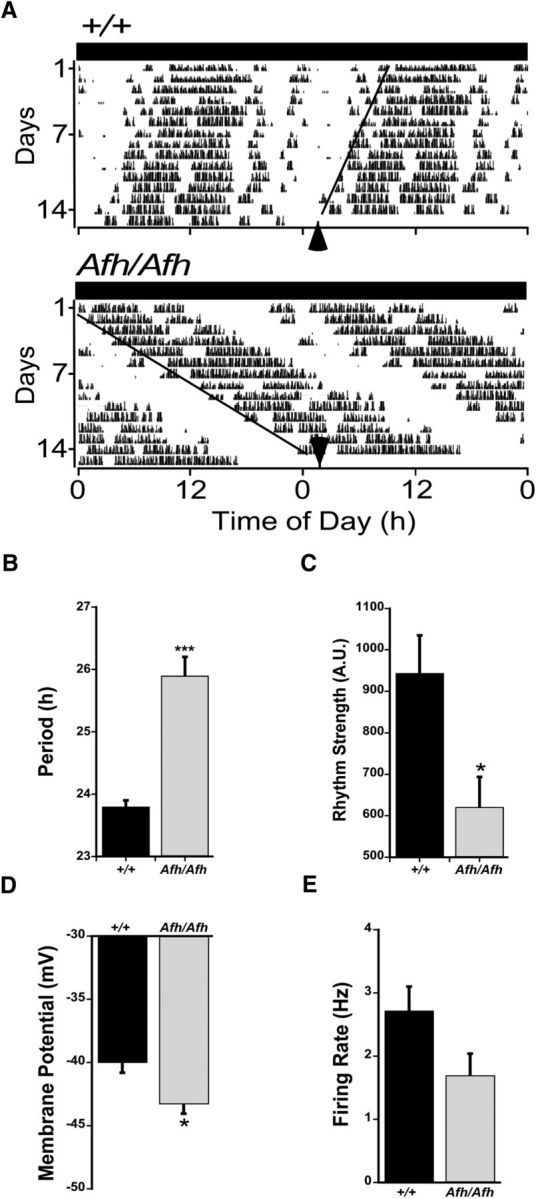
The hyperpolarized state of *Fbxl3^Afh/Afh^* vSCN neurons was sustained during the circadian night. ***A***, Examples of wheel-running activity (WRA) rhythms of *Fbxl3*^+/+^ (+/+) and *Fbxl3^Afh/Afh^* (*Afh/Afh*) mice in constant dark. WRA: CT12 = activity onset (black arrows). Circadian times of recorded cells were adjusted according to the circadian period of individual animals as calculated from activity onsets. The circadian period in WRA was lengthened by the *Afh* mutation (***B***), whereas rhythm strength was diminished (***C***). During circadian night, RMP was reduced by the *Afh* mutation (***D***), whereas spontaneous firing rate was slightly damped (***E***). Data in ***B***–***E*** are plotted as mean ± SEM. *p < 0.05, ***p < 0.001.

To determine how the *Afh* mutation influences the electrical states of SCN neurons, we performed *in vitro* whole-cell current-clamp recordings from dorsal (dSCN) and ventral (vSCN) cells in brain slices prepared from adult *Fbxl3^Afh/Afh^* and *Fbxl3*^+/+^ littermate mice that had been housed under 12 h LD conditions (Fig. 1*E* shows delineation of dSCN and vSCN subregions). Using well established criteria (Belle et al., 2009; Scott et al., 2010; Sakhi et al., 2014; Belle and Piggins, 2017), all recorded cells (*Fbxl3^Afh/Afh^*, *n* = 196 cells, ∼8 cells/slice; *Fbxl3*^+/+^, *n* = 213 cells, ∼7 cells/slice) were subsequently classified into one of four categories: (1) depolarized and silent (Fig. 1*A*); (2) depolarized and exhibiting low-amplitude membrane oscillations, DLAMO behavior (Fig. 1*B*); (3) spontaneously firing APs (Fig. 1*C*); and (4) hyperpolarized silent (Fig. 1*D*). For statistical analysis, depolarized cells of categories 1 and 2 were combined. No genotype-related differences were seen for parameters of these cell states; the RMP at which SCN neurons exhibited these states did not differ between *Fbxl3*^+/+^ and *Fbxl3^Afh/Afh^* animals (data not shown). In both genotypes, SCN cell state was significantly associated with time of day (day vs night: *Fbxl3*^+/+^ χ^2^ test = 8.532, *p* = 0.0014, *Fbxl3^Afh/Afh^* χ^2^ test = 25.3, *p* < 0.0001; Fig. 1*F*), with depolarized state less common and hyperpolarized states more frequent at night. At the subregional level, cell state was significantly associated with time of day (day vs night) in both the dSCN (χ^2^ test = 6.374, *p* = 0.041) and vSCN (χ^2^ test = 21.21, *p* < 0.0001) of *Fbxl3^Afh/Afh^* mice, but only vSCN of *Fbxl3*^+/+^ animals (χ^2^ test = 7.1, *p* = 0.028). At night, neuronal state expression differed between dSCN and vSCN of *Fbxl3^Afh/Afh^* animals (χ^2^ test = 9.75, *p* = 0.008), but not *Fbxl3*^+/+^ mice (*p* > 0.05). No subregional differences in neuronal state expression were detected during the day in either genotype.

To assess whether the *Afh* mutation influenced temporal expression of RMP, SFR, and *R*_input_ of SCN neurons, data from cells of each genotype were allocated according to the time of recording to one of four 6 h time bins corresponding to early day (ZT0–ZT6), late day (ZT6–ZT12), early night (ZT12–ZT18), and late night (ZT18–ZT24). The parameters of these properties were then compared in the vSCN (*Fbxl3^Afh/Afh^*, *n* = 137 cells; *Fbxl3*^+/+^, *n* = 140 cells) or dSCN (*Fbxl3^Afh/Afh^*, *n* = 59 cells; *Fbxl3*^+/+^, *n* = 73 cells), respectively. For RMP in the vSCN, two-way ANOVA revealed a significant effect of genotype (*F*_(1,269)_ = 8.3984, *p* = 0.004), time of day (*F*_(3,269)_ = 16.62, *p* < 0.0001) and genotype × time of day interaction (*F*_(3,269)_ = 3.1569, *p* = 0.026). Planned single degree of freedom contrasts indicated significant day to night differences in the RMP of both *Fbxl3^Afh/Afh^* and *Fbxl3*^+/+^ vSCN neurons (*F*_(1,269)_ = 47.65, *p* < 0.0001 and *F*_(1,269)_ = 7.70, *p* = 0.0083, respectively). During the early or late day, there were no intergenotype differences (single degree contrasts, *p* > 0.05), whereas at night, *Fbxl3^Afh/Afh^* neurons were more hyperpolarized than their *Fbxl3*^+/+^ counterparts at both ZT12–ZT18 (*F*_(1,269)_ = 8.74, *p* = 0.0083) and ZT18–ZT24 (*F*_(1,269)_ = 12.64, *p* = 0.0004; Fig. 1*G*). Such genotype differences in RMP were not detected in the dSCN where two-way ANOVA indicated a significant time of day effect only (*F*_(3,124)_ = 8.25, *p* < 0.0001). These findings reveal that the influence of the *Afh* mutation on the level of RMP is readily detectable in the vSCN, but not the dSCN. Further, the *Afh* mutation results in enhanced hyperpolarization of vSCN neurons throughout the night.

For analysis of SFR, depolarized cells were excluded as they cannot discharge APs. No genotype differences were detected in the vSCN, and consequently data were collapsed across genotypes; two-way ANOVA revealed a significant time of day effect (*F*_(3,232)_ = 9.63, *p* < 0.0001), with a significant day/night difference (2.7 ± 0.2 Hz vs 1.7 ± 0.1 Hz, *F*_(1,232)_ = 13.1, *p* = 0.0004; data not shown). In the dSCN, no significant effects on SFR were detected (*F* ratios, *p* > 0.05). For *R*_input_, data from cells in all states were used and in the vSCN and dSCN, no significant differences were detected, although in both SCN subregions, genotype differences approached significance (*p* < 0.08 and 0.09, respectively). Therefore, the effects of the *Afh* mutation were most overtly expressed as a nocturnal decrease in RMP in the vSCN.

To ascertain with greater temporal resolution how alterations in the stability of CRY proteins influenced SCN neuronal properties, cubic spline functions were fit to the RMP and SFR data from the vSCN and dSCN recordings and the times of the peak and nadir in these measures of SCN neuronal excitability determined (Fig. 2). For vSCN neurons of *Fbxl3*^+/+^ mice, maximal depolarized RMP (mean ± 95% confidence interval, −38 mV; 95% confidence interval −36.3 to −39.8 mV) occurred at ZT3.9 and hyperpolarized (−42.2 mV; confidence interval −40.6 to −43.8 mV) at ZT16 (Fig. 2*A*,*B*). For *Fbxl3^Afh/Afh^* vSCN neurons, the peak and nadir values were delayed by 2.3 and 1.2 h, respectively, with the mean maximal depolarized (−37.5 mV; confidence interval −35.2 to −39.9 mV) state occurring at ZT6.2 and mean maximal hyperpolarized state (−48.2 mV; confidence interval −46.2 to −50.3 mV) at ZT17.2 (Fig. 2*A*,*B*). Consistent with the statistical analysis above, the night-time was when a clear genotype-related difference was seen with vSCN *Fbxl3^Afh/Afh^* neurons more hyperpolarized than their *Fbxl3*^+/+^counterparts for most of the projected night. Comparison of the fitted splines with the GLIMMIX procedure indicated that from ZT14–ZT22, there were significant differences between the RMP of *Fbxl3^Afh/Afh^* neurons and that of *Fbxl3*^+/+^ cells (range *p* < 0.05–*p* < 0.001). This can be visualized in the plotted mean ± 95% confidence interval of each genotype's spline as the absence of an overlap throughout the majority of night phase (Fig. 2*A*). Indeed, a higher amplitude (peak-nadir difference) rhythm in RMP was seen for *Fbxl3^Afh/Afh^* (∼10.7 mV) compared with *Fbxl3*^+/+^ vSCN (∼4.2 mV) neurons. Further, the time required to ascend from nadir to peak RMP or to descend from peak to nadir RMP differed between the two genotypes. For the *Fbxl3^Afh/Afh^* vSCN, it took ∼13.3 h from the nadir to achieve peak RMP, but only ∼10.7 h to decline from this peak to the nadir in RMP (Fig. 2*B*). This asymmetry is not seen in the *Fbxl3*^+/+^ vSCN, where ∼11.9 h are required to rise from the nadir to peak RMP and ∼12.1 h to decline from the peak RMP to the nadir (Fig. 2*B*). Therefore, delaying degradation of CRYs alters the rate of change of RMP, with *Fbxl3^Afh/Afh^* vSCN increasing by ∼0.8 mV/h and decreasing by ∼1 mV/h, whereas *Fbxl3*^+/+^ vSCN neurons increase and decrease at similar rate of ∼0.35 mV/h. For SFR in the vSCN, peak and nadir measures of firing frequency (Hz) did not differ between the genotypes, but broadly consistent with RMP, peak daytime firing rate was delayed by ∼2 h and the nocturnal nadir delayed by ∼0.9 h in *Fbxl3^Afh/Afh^* mice (Fig. 2*C*). Similarly, in the dSCN, the timing of the peak and nadir in RMP was delayed in the *Fbxl3^Afh/Afh^* SCN (∼1.3 and 2.8 h, respectively; Fig. 2*D*), with the amplitude in this rhythm resembling the genotype-related differences seen in the vSCN (∼10.4 mV in *Fbxl3^Afh/Afh^* dSCN and ∼5.1 mV in *Fbxl3*^+/+^ dSCN; Fig. 2*E*). Interestingly, *Fbxl3*^+/+^ dSCN neurons exhibited asymmetry in their RMP, with the rise from nadir (∼−41.4 mV) to peak (∼−39.1 mV) taking 12.7 h, whereas the decline from peak to trough took ∼11.3 h (Fig. 2*E*). This asymmetry was reversed in the *Fbxl3^Afh/Afh^* SCN, with the decline from peak to trough taking ∼12.8 and ∼11.2 h elapsing in rise from trough to peak. For SFR in the dSCN, no prominent genotype differences were observed, with the timing of the peak in firing advanced (∼0.2 h) and the trough delayed (∼0.3 h) in the *Fbxl3^Afh/Afh^* animals (Fig. 2*F*). However, the amplitude of the SFR rhythm in *Fbxl3^Afh/Afh^* recordings was damped (∼0.4 Hz) compared with that of *Fbxl3*^+/+^ dSCN (∼1.4 Hz). These findings reveal an unexpected consequence of the *Fbxl3^Afh^* mutation that is manifested as larger daily excursions and asymmetrical rates of change in RMP of vSCN neurons as well as altered symmetry in daily variation in RMP of dSCN neurons.

### Alterations in electrophysiological activity of Fbxl3*^Afh/Afh^* SCN neurons are independent of the light/dark cycle

The environmental LD cycle synchronizes the SCN circadian clock to the external world with light information conveyed directly to the SCN via the glutamatergic retinohypothalamic tract (Ebling, 1996; Brown, 2016). It is possible that constraining the long period (∼26 h) of the *Fbxl3^Afh/Afh^* SCN circadian pacemaker to 24 h disrupts daily expression of SCN neuronal states. To determine whether the hyperpolarized neuronal states expressed during the late night in the *Fbxl3^Afh/Afh^* vSCN persist in the absence of the LD cycle, we made recordings from brain slices prepared from *Fbxl3*^+/+^ and *Fbxl3^Afh/Afh^* mice that had been free-running in DD for 8–14 d. Under DD conditions, *Fbxl3^Afh/Afh^* mice (*n* = 8) expressed a longer intrinsic period in wheel-running than *Fbxl3*^+/+^ (*n* = 8) counterparts (25.9 ± 0.3 vs 23.8 ± 0.1 h; *t*_(8.5)_ = 6.64, *p* = 0.0001; Fig. 3*A*,*B*) and the strength of this rhythm was reduced compared with that of *Fbxl3*^+/+^ animals (620 ± 74 vs 944 ± 91 A.U.; *t*_(14)_ = 2.76 *p* = 0.015; Fig. 3*C*). For animals in DD, CT12 is the time of activity onset and times of recording were plotted with respect to CT12 (Fig. 3*A*, black arrows), with all recording time points adjusted with respect to the free-running period of the individual animal. The mean RMP of *Fbxl3^Afh/Afh^* vSCN neurons at late circadian night (Fig. 3*D*; −43.2 ± 1.1 mV, *n* = 32; 3–5 cells per slice) was significantly more hyperpolarized than *Fbxl3*^+/+^ counterparts (−40 ± 0.8 mV, *n* = 22; 2–4 cells per slice; *t*_(51.29)_ = 2.35, *p* = 0.022)., reflecting in part a slight increase in the prevalence of hyperpolarized cells in *Fbxl3^Afh/Afh^* vSCN at this time (8/32 cells compared with 2/24 cells in *Fbxl3*^+/+^ vSCN). At this late-night phase, the SFR of *Fbxl3^Afh/Afh^* vSCN neurons also tended to be lower than that of *Fbxl3*^+/+^ vSCN cells (1.5 ± 0.3 Hz *Fbxl3^Afh/Afh^* vs 2.4 ± 0.4 Hz *Fbxl3*^+/+^; *t* test, *p* = 0.11; Fig. 3*E*). These results reveal that the reduced night-time excitability of *Fbxl3^Afh/Afh^* vSCN neurons is sustained in the absence of the LD cycle.

### Intrinsic and network influences on SCN neuronal activity

In genetically and neurochemically intact SCN, intrinsic TTFL control of cellular excitability results in pronounced neuronal state-related differences in *R*_input_ (Belle et al., 2009, 2014). To assess whether the reduced excitability of *Fbxl3^Afh/Afh^* vSCN neurons during the late night arose from alterations in the intrinsic TTFL drive or extrinsically through changes in synaptic activity of the SCN network, we compared *R*_input_ in spontaneously firing as well as hyperpolarized silent neurons in the *Fbxl3^Afh/Afh^* (*n* = 76) and *Fbxl3*^+/+^ vSCN at night (*n* = 65). In *Fbxl3^Afh/Afh^* vSCN neurons, *R*_input_ did not vary between these two cell states (2.1 ± 0.1 vs 1.8 ± 0.3 GΩ; *t* test, *p* = >0.05), whereas in *Fbxl3*^+/+^ vSCN neurons, *R*_input_ was significantly higher in firing cells compared with hyperpolarized silent cells (2.3 ± 0.1 vs 1.3 ± 0.2 GΩ; *t*_(74)_ = 4.74, *p* = 0.008; data not shown). These findings indicate that the significant intrinsic modulation of neuronal state recorded in *Fbxl3*^+/+^ vSCN neurons is diminished in *Fbxl3^Afh/Afh^* vSCN neurons. Therefore, changes in extrinsic SCN network synaptic activity more likely underpin the more hyperpolarized states of *Fbxl3^Afh/Afh^* vSCN neurons at night.

**Figure 4.**
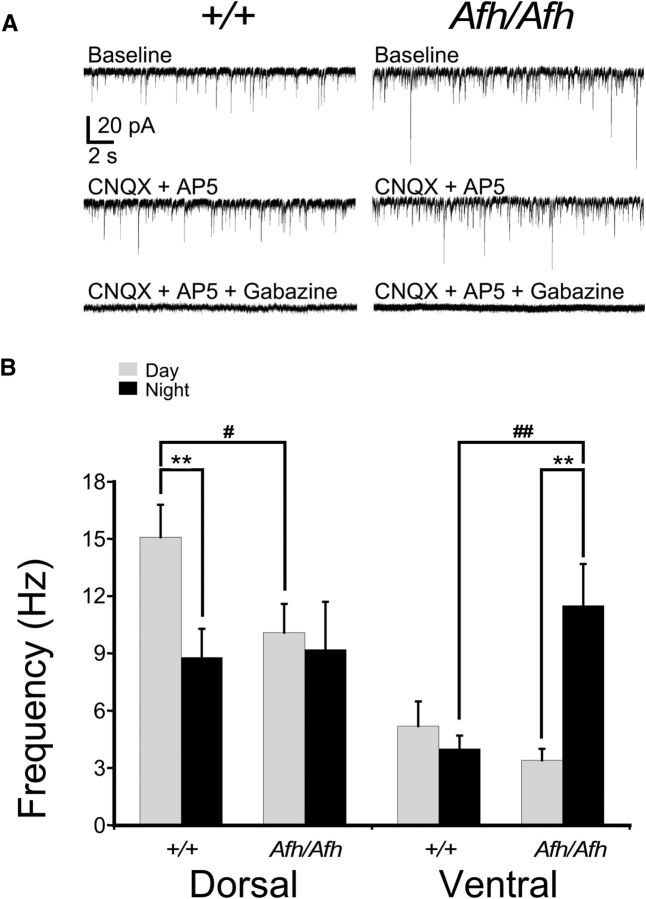
The *Fbxl3^Afh^* mutation elevates the frequency of GABAergic synaptic events in vSCN neurons during the night. In voltage-clamp recordings made at a holding potential of −70 mV, spontaneous synaptic events present in SCN slices from *Fbxl3*^+/+^ (+/+) and *Fbxl3^Afh/Afh^* (*Afh/Afh*) mice (***A***, top traces) were unaffected by glutamatergic antagonists (CNQX blocks AMPA receptors, AP-5 blocks *N*-methyl-D-aspartate (NMDA) receptors; ***A***, middle traces), but abolished by the addition of the GABA antagonist, gabazine (***A***, bottom traces). This indicates that GABAergic and not glutamatergic sPSCs, are predominant in the SCN of *Fbxl3*^+/+^ and *Fbxl3^Afh/Afh^* animals. ***B***, Compared with the night, the frequency of the GABAergic sPSCs was significantly higher during the day in the dSCN but not the vSCN of *Fbxl3*^+/+^ mice. By contrast, in the vSCN of *Fbxl3^Afh/Afh^* mice, the night-time frequency of the GABAergic sPSCs was significantly higher than that observed during the day, and significantly higher than that recorded in the vSCN of *Fbxl3*^+/+^ mice at this time. Further the daytime frequency of GABAergic sPSCs in the dSCN of *Fbxl3^Afh/Afh^* animals was significantly lower than that recorded during the day in the dSCN of *Fbxl3*^+/+^ mice. Data in ***B*** are plotted as mean ± SEM. Within-genotype differences are denoted by **p < 0.01, and between-genotype differences denoted by #*p* < 0.05, ##*p* < 0.01.

To gain further insight into potential SCN network alterations in the *Fbxl3^Afh/Afh^*, we examined GABAergic transmission since most neurons in the vSCN and dSCN contain GABA (Buijs et al., 1994; Castel and Morris, 2000; Moore, 2013) and because many ionotropic GABA_A_ receptor subunits are present throughout the SCN (Gao et al., 1995; O'Hara et al., 1995; Albers et al., 2017). In sample voltage-clamp recordings made across late day/early night, we found that in both dSCN and vSCN subregions of *Fbxl3*^+/+^ (*n* = 4 cells each subregion) and *Fbxl3^Afh/Afh^* (*n* = 4 cells each subregion) mice, glutamatergic antagonists CNQX (50 μm) and AP-5 (50 μm) did not alter the frequency of sPSCs, whereas the GABA_A_ receptor antagonist, gabazine (20 μm), abolished them (Fig. 4*A*). This confirms that GABA-GABA_A_ receptor signaling is prevalent throughout this structure (Itri et al., 2004; Scott et al., 2010; Belle et al., 2014). The frequency, but not the amplitude of such GABAergic events in the genetically intact murine SCN varies from day to night (Itri et al., 2004) and to evaluate whether the *Afh* mutation altered this temporal variation, we made voltage-clamp recordings from cells sampled in dSCN and vSCN of *Fbxl3^Afh/Afh^* and *Fbxl3*^+/+^ brain slices around the late day (ZT6–ZT10) or middle of the night (ZT15–ZT21) phase. Consistent with previous research on genetically intact mouse SCN (Itri et al., 2004), the frequencies of synaptic inputs recorded in *Fbxl3*^+/+^ slices were robustly higher in the dSCN during the day (15.1 ± 1.7 Hz, *n* = 16) than at night (8.8 ± 1.5 Hz, *n* = 16, *t* test = 2.78, df = 30, *p* = 0.0093), whereas no such day/night difference was seen in the vSCN (5.2 ± 1.3 Hz, *n* = 14, and 4.0 ± 0.7 Hz, *n* = 20, respectively; *t* test, *p* > 0.05; Fig. 4*B*). By contrast, in *Fbxl3^Afh/Afh^* slices, there was no day/night difference in the dSCN (10.1 ± 1.5 Hz, *n* = 15 and 9.2 ± 2.5 Hz, *n* = 13, respectively; *t* test *p* > 0.05), but in the vSCN, the frequency of GABAergic sPSCs was significantly higher at night (11.5 ± 2.2, *n* = 18) than during the day (3.4 ± 0.6, *n* = 15; *t*_(19.5)_ = 3.55, *p* = 0.0021; Fig. 4*B*). Indeed, this elevated night-time activity was also significantly higher than in the *Fbxl3*^+/+^ vSCN at this time (*t*_(20.4)_ = 20.43, *p* = 0.0039), whereas the daytime frequency of GABAergic PSCs in the *Fbxl3^Afh/Afh^* dSCN differed from that of the *Fbxl3*^+/+^ dSCN at this time (*t*_(29)_ = 2.19, *p* = 0.018). This indicates that *Fbxl3^Afh^* mutation unexpectedly differentially changes temporal and regional GABAergic signaling in the SCN. Therefore, enhanced GABAergic inputs at night accompany the reduced nocturnal RMP of *Afh* vSCN neurons.

### GABA_A_ receptor blockade destabilizes the network in Fbxl3^+/+^ but not Fbxl3*^Afh/Afh^* SCN

**Figure 5.**
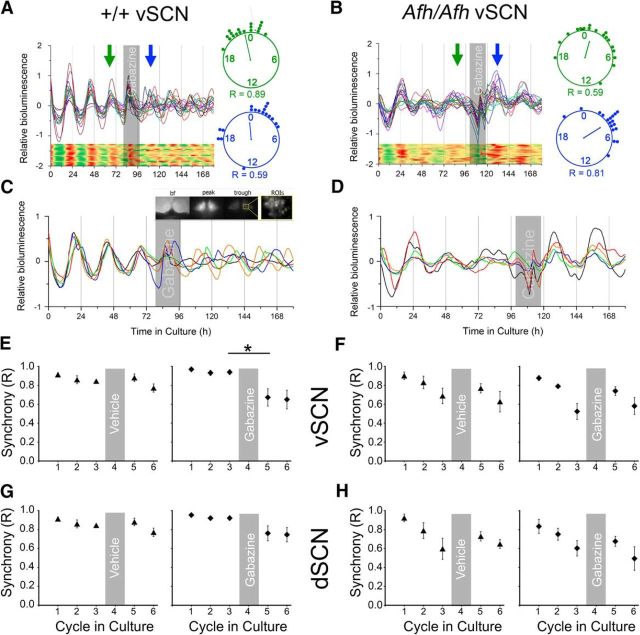
Blockade of GABA signaling fails to desynchronize PER2::LUC single-cell rhythms in the *Fbxl3^Afh/Afh^* SCN. PER2::LUC single-cell bioluminescence recordings discriminated in *Fbxl3*^+/+^ (+/+; ***A***, ***C***, ***E***, ***G***) and *Fbxl3^Afh/Afh^* (*Afh/Afh*; ***B***, ***D***, ***F***, ***H***) SCN slice preparations. From each slice 18–22 cell-like ROIs were discriminated from the dSCN and vSCN subregions. Synchrony (*R*) was assessed before (cycles 1–3) and following treatment (cycles 5–6), and single-cell heatmaps of the representative traces are shown for better visualization (***A*** and ***B*** show data from vSCN). Gray shaded areas depict time of treatment. Rayleigh plots and *R* values are shown for cycles 3 (green) and 4 (blue) of the representative traces of *Fbxl3*^+/+^ (***A***) and *Fbxl3^Afh/Afh^* (***B***) genotypes, with the clock hands depicting the mean peak time and their length representing the *R* value (the longer the clock hand, the larger the *R* value). ***C***, ***D***, Recordings of five cells from a vSCN slice of each genotype are shown in magnification and example bf, peak, and trough bioluminescence images from *Fbxl3*^+/+^ vSCN are depicted in ***C*** (inset). Gabazine was detrimental to synchrony in the *Fbxl3*^+/+^ vSCN, whereas vehicle treatment was not (***C***, ***E***). Neither vehicle nor gabazine had significant effects on cell synchrony in the *Fbxl3*^+/+^ dSCN (***G***). The desynchronizing action of gabazine was not detected in the *Fbxl3^Afh/Afh^* vSCN and dSCN (***D***, ***F***, ***H***). Vehicle treatment of the SCN was without effect on synchrony in either vSCN (***F***) or dSCN (***H***) of the *Fbxl3^Afh/Afh^* SCN tissue slices. Data in ***E***–***H*** are plotted as mean ± SEM. *p < 0.05.

Robust long-term timekeeping within the SCN depends on the synchrony among its cell autonomous oscillators (Yamaguchi et al., 2003). GABA signaling influences SCN cellular synchrony (DeWoskin et al., 2015) and our observation that acute GABAergic activity in the SCN is altered by the *Afh* mutation led us to speculate that this could destabilize the SCN network. To test this, we looked for longer-term influences on timekeeping properties of the SCN by monitoring the expression of a bioluminescent reporter of the molecular clock protein, PER2::LUC at the single-cell level in SCN brain slices over several days in culture (Fig. 5). We then determined how blockade of GABA-GABA_A_ receptor signaling with gabazine influenced these SCN cellular rhythms. In agreement with behavioral data, the dSCN and vSCN in slices (*n* = 9) from *Fbxl3^Afh/Afh^* mice oscillated with longer periods than that seen in slices (*n* = 8) prepared from *Fbxl3*^+/+^ animals (dSCN: 25.4 ± 0.3 vs 23.7 ± 0.1 h'; *t*_(9.74)_ = 5.38, *p* = 0.0003; vSCN: 26.1 ± 0.5 vs 23.9 ± 0.2 h; *t*_(10.46)_ = 4.09, *p* = 0.002). Consistent with the our electrophysiological results, we also observed that between the second and third cycle in culture, the time taken to rise from nadir to peak in the global PER2::LUC bioluminescence signal of the whole SCN slice was elongated ∼3.9 h by the *Afh* mutation (*Fbxl3*^+/+^: 12.4 ± 0.27 vs *Fblx3^Afh/Afh^*: 16.3 ± 0.52 h; *t*_(12)_ = 6.7, *p* < 0.0001), whereas the time elapsing from peak to trough was shortened by ∼1.4 h (*Fbxl3*^+/+^: 12.45 ± 0.25 vs *Fbxl3^Afh/Afh^*: 11.1 ± 0.41 h; *t*_(15)_ = −2.84, *p* = 0.016). This indicates that the *Afh* mutation asymmetrically alters molecular clock oscillations.

Genetically and neurochemically intact SCN networks remain synchronized over several days in culture (Yamaguchi et al., 2003; Evans et al., 2013) and in agreement with our previous research (Guilding et al., 2013), we found that within three circadian cycles *in vitro*, single cells of the dSCN and vSCN of *Fbxl3^Afh/Afh^* mice became much less synchronized than their *Fbxl3*^+/+^ dSCN counterparts. For the dSCN, two-way ANOVA indicated significant effects of matching subjects (*F*_(14,28)_ = 4.93, *p* = 0.0002), time (*F*_(2,28)_ = 23.81, *p* < 0.0001), genotype (*F*_(1,14)_ = 9.45, *p* = 0.0083), and time × genotype interaction (*F*_(2,28)_ = 13.59, *p* < 0.0001) with significant genotype differences in cellular synchrony occurring on cycle 3 (Sidak *post hoc* test, *p* < 0.0001). In the vSCN, two-way ANOVA revealed significant effects of matching subjects (*F*_(14,28)_ = 3.3, *p* = 0.0035), time (*F*_(2,28)_ = 18.17, *p* < 0.0001), genotype (*F*_(1,14)_ = 20.24, *p* = 0.0005), and time × genotype interaction (*F*_(2,28)_ = 11.3, *p* = 0.0003), with significant genotype differences in cellular synchrony occurring on cycles 2 and 3 (Sidak tests, *p* = 0.034 and *p* < 0.0001, respectively). Next we assessed how GABA_A_ blockade with gabazine subsequently influenced slice rhythms and cellular synchrony. On day 4 in culture, a change to new control media did not overtly affect synchrony in the dSCN or vSCN of *Fbxl3*^+/+^ mice, whereas treatment with media containing gabazine (20 μm) subsequently desynchronized the cells in the vSCN (*t*_(4)_ = 3.92, *p* = 0.017), but not in the dSCN (*t* test *p* > 0.05; Fig. 5*A*,*C*,*E*,*G*). By contrast, the effects of gabazine treatment (20 μm) on *Fbxl3^Afh/Afh^* SCN slices were much less pronounced, with no differences between responses to vehicle and gabazine treatment; indeed vSCN and dSCN networks maintained synchrony following both treatments (*t* tests, all *p* > 0.05; Fig. 5*B*,*D*,*F*,*H*).

### The Fbxl3*^Afh/Afh^* SCN network shows enhanced phase-advances to AMPA

**Figure 6.**
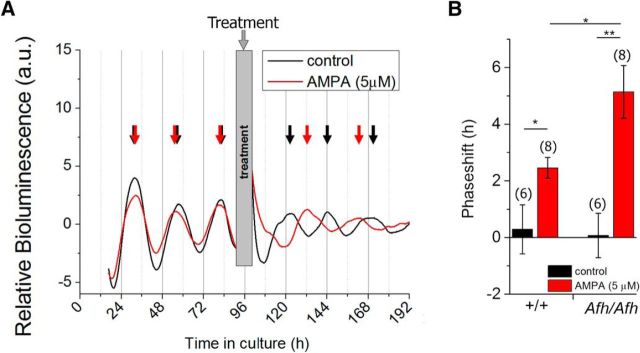
Phase-advancing effects of AMPA are enhanced in the *Fbxl3^Afh/Afh^* SCN. ***A***, Example traces of photomultiplier tube bioluminescence recordings from an *Fbxl3^Afh/Afh^* (*Afh/Afh*) SCN explant treated with vehicle (black trace) or 5 μm AMPA (red trace) at the trough of day 5 in culture. The arrows indicate the point of peak expression used for calculation of the period, with red arrows delineating peaks pre- and post-AMPA treatment and black arrows showing peaks prevehicle and postvehicle control treatment. ***B***, Compared with vehicle treatment, AMPA evoked significant phase advance in the timing of peak PER2::LUC bioluminescence in both *Fbxl3*^+/+^ (+/+) and *Fbxl3^Afh/Afh^* SCN explants. Larger advances were evoked by AMPA in *Fbxl3^Afh/Afh^* compared with *Fbxl3*^+/+^ SCN explants (5.1 ± 0.9 vs 2.5 ± 0.4 h, *p* = 0.0018), sample sizes given in brackets. *p < 0.05, **p < 0.01.

The functional implications for reduced synchrony in the *Fbxl3^Afh/Afh^* SCN slices are unclear. Previously we reported that during the circadian night, transient exposure to light evokes much larger phase shifts in wheel-running rhythms of *Fbxl3^Afh/Afh^* mice than *Fbxl3*^+/+^ animals (Guilding et al., 2013). The basis for this is unknown, but because at the light-sensitive night phase *Fbxl3^Afh/Afh^* vSCN neurons are more hyperpolarized than their *Fbxl3*^+/+^ counterparts, we investigated the potential contribution of the SCN to this enhanced light responsiveness. To do this, we used luminometry (in which the global photon output of the slice is measured but not visualized as an image), and assessed how rhythms in PER2::LUC bioluminescence in SCN brain slices were reset by a neurochemical mimic of the light input pathway, the glutamatergic agonist AMPA (5 μm) given at the nocturnal nadir of this rhythm. In both genotypes, AMPA treatment evoked phase-shifts in the timing of peak bioluminescence that were significantly advanced from that observed with vehicle treatment (*Fbxl3*^+/+^: *t*_(12)_ = 2.59, *p* = 0.024; *Fbxl3^Afh/Afh^*: *t*_(12)_ = 3.98, *p* = 0.0018). Advances evoked by AMPA were significantly larger in *Fbxl3^Afh/Afh^* compared with *Fbxl3*^+/+^ SCN slices (5.1 ± 0.9 vs 2.5 ± 0.4 h; *t*_(9.12)_ = 2.64, *p* = 0.013; Fig. 6*A*,*B*). Therefore, the functional implication of the altered cellular state and less synchronized network in the SCN of *Fbxl3^Afh/Afh^* mice is an increased readiness to phase-shift in response to an excitatory mimic of the light input pathway during the projected night.

## Discussion

Here we show for the first time how reduction of function in Fbxl3-mediated ubiquitination and subsequent targeting for proteosomal degradation of key repressors of the molecular clock affects the daily dynamics in SCN neuronal membrane excitability. The *Fbxl3^Afh^* mutation unexpectedly diminishes TTFL coordination of neuronal state, in part seceding this regulation at night to elevate GABA signals in the vSCN. These signals possibly come from a rewiring of the SCN network and lead to hyperpolarization in the vSCN. Consequently, the capability of vSCN neurons to maintain synchrony is reduced and SCN resetting responses to a glutamatergic mimic of the light input pathway during the projected night are enhanced. These results substantiate evidence from several laboratories of the importance of post-translational modification and the SCF^Fbx^ complex to circadian clock function (Dardente et al., 2008; Hirano et al., 2013; Yoo et al., 2013; Zhao et al., 2016). They extend our earlier finding of diminished synchrony in the *Fbxl3^Afh^* SCN and indicate that the enhanced resetting of *Fbxl3^Afh/Afh^* mice to light that we reported earlier (Guilding et al., 2013) is in part mediated at the level of the SCN. This indicates hitherto unexpected consequences of diminution in SCF^Fbxl3^ activity on neuronal state, network organization, and GABA function in the vSCN.

GABAergic signaling contributes to synchrony in neural networks throughout the vertebrate brain (Mann and Paulsen, 2007; Uhlhaas and Singer, 2010; Cea-Del Rio and Huntsman, 2014; Khazipov, 2016). Here, consistent with previous work (Freeman et al., 2013), in the genetically and neurochemically intact *Fbxl3*^+/+^ SCN, GABA-GABA_A_ communication facilitates neuronal synchrony such that pharmacological blockade of this signal with gabazine impairs clock cell synchrony in the vSCN. However, in the vSCN of *Fbxl3^Afh/Afh^* mice, where we find that GABAergic synaptic activity is unusually elevated and cell autonomous oscillators are prone to desynchronize, gabazine does not further diminish synchrony in cell oscillations. This suggests that in the genetically weakened SCN, GABA-GABA_A_ receptor signaling does not play a prominent role in promoting synchrony. At present, it is unclear why this is the case, but it could arise through alterations in the polarity of GABA's actions. Indeed, tension between excitatory and inhibitory GABA can desynchronize SCN neuronal oscillators (DeWoskin et al., 2015; Myung et al., 2015; Azzi et al., 2017; for review, see Albers et al., 2017). Thus, the prolongation in CRY stability alters the day/night variation in GABA signaling, which may counteract this neurochemical's role in promoting synchrony among *Flxl3^Afh/Afh^* vSCN clock cells.

Our findings are also consistent with the prevailing view that there are potentially distinct roles and functions for different subregions of the SCN (Albus et al., 2005; Brown and Piggins, 2009; Enoki et al., 2012; Evans et al., 2013; Azzi et al., 2014). Here we targeted an exclusive dSCN and more inclusive vSCN subregion (Fig. 1). Neurochemically, this dSCN region corresponds to an area characterized by arginine vasopressin neurons, whereas the vSCN is contains a range of neuropeptide-synthesizing cells including those that contain one or a combination of vasoactive intestinal polypeptide or arginine vasopressin or gastrin-releasing peptide (Antle and Silver, 2005; Moore, 2013). Our data suggest that although SCF*^Afh^* elongates the period of molecular and neurophysiological events in the dSCN, it is the vSCN neurons where these and other effects are most pronounced. Previous investigations have reported that the vSCN subregion is more responsive and malleable to environmental cues than the dSCN (Brown and Piggins, 2009). Our findings extend this to raise the possibility that vSCN neurons are also more susceptible to genetic perturbation in the intracellular environment such as that arising from compromising the SCF^Fbxl3^ post-translational modification mechanism. The enhanced GABA release at night resulted in more hyperpolarized neuronal states, thereby heightening the daily rhythm in RMP; an effect not detected in the dSCN. However, the daily rhythm in spontaneous firing rate is somewhat reduced in the dSCN but not the vSCN, indicating subregional heterogeneity in the effects of Fbxl3^Afh^ on the relationship between RMP and AP firing. Because firing rate of dSCN neurons is believed to represent the output of the SCN circadian pacemaker, this could contribute to the reduced circadian control of behavioral rhythms noted previously in *Fbxl3^Afh/Afh^* mice (Guilding et al., 2013). One caveat is that we cannot explain why elevated GABAergic events recorded during the day in the dSCN of *Fbxl3*^+/+^ mice are not accompanied by suppressed RMP.

To date, assessment of post-translational modification on SCN neuronal activity has been mostly limited to recording the daily profile in spontaneous firing rate of rodents in which the *tau* mutation in casein kinase 1 ε (Ck1ε) accelerates phosphorylation of PER proteins and their targeting for proteosomal degradation (Gallego et al., 2006). This gain-of-function mutation accelerates the TTFL clock and shortens the circadian period in behavioral rhythms to ∼20 h (Ralph and Menaker, 1988; Meng et al., 2008). In the SCN, this period shortening effect is seen as an advance of the daily peak and nadir of SCN spontaneous firing rate, but the amplitude of this rhythm appears unaffected (Davies and Mason, 1994; Liu et al., 1997; Meng et al., 2008). Here in a mouse with hypomorphic Fbxl3, we find that the peak and nadir in spontaneous firing rate of vSCN neurons is predictably delayed and that the amplitude of this rhythm is unchanged, whereas in the dSCN, the timing of the firing rate rhythm is not notably altered, but the amplitude is reduced. The daily rhythm in RMP in the dSCN and vSCN of *Fbxl3^Afh/Afh^* animals is also delayed and its amplitude in the vSCN is unexpectedly increased through nocturnally elevated GABA activity. Current evidence indicates that Fbxl3 exerts most influence on CRY degradation at the level of the nucleus (Busino et al., 2007; Hirano et al., 2013; St John et al., 2014) and this raises the possibility that Fbxl3^Afh^ prolongation of CRYs' actions enhances transcription of factors promoting GABAergic signaling. Further the daily variation in intrinsic drive on vSCN neuronal excitability (as measured by *R*_input_) is reduced by hypomorphic Fbxl3, suggesting altered temporal expression of ion channels by this change in dynamic control of intracellular levels of the CRYs. Because Fbxl3's circadian actions may preferentially affect CRY1 over CRY2 (Anand et al., 2013), it will be interesting in future studies to differentiate the targets of the prolonged stabilization of CRY1 compared with those of CRY2.

These findings also point to how delaying Fbxl3-mediated ubiquitination and targeting for proteosomal degradation of CRYs and prolonged transcriptional repression (St John et al., 2014) leads to asymmetry in the daily variation in RMP (and to a lesser extent spontaneous firing rate) in the SCN. Intriguingly, this temporal elongation in nadir-to-peak RMP broadly resembles that noted for the daily rhythm in PER2::LUC expression (Godinho et al., 2007; Anand et al., 2013) in the *Fbxl3^Afh/Afh^* mouse SCN. In those investigations, the rate of increase in PER2::LUC expression from the nadir to the peak in *Fbxl3^Afh/Afh^* SCN was lower than that of +/+ mice, thereby delaying the time at which peak PER2::LUC expression was achieved. Indeed, in the present investigation, we also find that in the *Fbxl3^Afh/Afh^* mouse SCN, the time taken to rise from trough-to-peak in PER2::LUC expression is extended by ∼3.9 h over that of the *Fbxl3*^+/+^ SCN, whereas that of the decline from peak-to-trough is reduced by ∼1.4 h. Together, these findings suggest that although the general correlation between clock gene expression and neuronal activity is maintained in *Fbxl3^Afh/Afh^* mice, intrinsic TTFL control of neuronal state is surprisingly diminished and instead, the contribution of intercellular signaling to SCN neuronal state is increased. Consistent with our previous research (Belle et al., 2009), we find that RMP provides better insight into the relationship between the TTFL and SCN neurons. *In vivo*, expression of most clock genes/proteins is markedly reduced in the SCN of Fbxl3^Afh^ (Godinho et al., 2007) and Fbxl3^Ovtm^ (Siepka et al., 2007; Yoo et al., 2013) animals, but these mice maintain rhythms (albeit weakened) in wheel-running behavior in constant dark. This indicates that *in vivo*, other signals such as feedback from behavioral state (Hughes and Piggins, 2012) act to maintain a critical level of SCN synchrony and function in these animal models.

In summary, we found that an alteration in post-translational modification of essential repressor components of the molecular circadian clock asymmetrically alters the daily profile in neuronal excitability and unexpectedly increases the amplitude in the daily rhythm in membrane excitability. A diminution in intrinsic molecular clock influence on neuronal state and an increase in GABAergic synaptic activity underpin this enhancement in the circadian profile of neuronal excitability. Such alterations contribute to a reduction in the ability of cell autonomous oscillators in the vSCN to maintain synchrony that in turn increases the magnitude of resetting to a physiologically relevant excitatory input signal. These findings confirm the vital contribution of post-translational modification and in particular Fbox-mediated proteosomal degradation to circadian timekeeping. This further extends our knowledge by revealing unexpected consequences of perturbing SCF^flbxl3^ on intrinsic cell state and neural network communication.
